# Caregivers of cancer patients: what are their information-seeking behaviours and resource preferences?

**DOI:** 10.3332/ecancer.2020.1068

**Published:** 2020-07-10

**Authors:** Gek Phin Chua, Quan Sing Ng, Hiang Khoon Tan, Whee Sze Ong

**Affiliations:** 1 Cancer Education and Information Service (Research & Data), National Cancer Centre Singapore, 169610 Singapore; 2 Division of Medical Oncology, National Cancer Centre Singapore, 169610 Singapore; 3 Division of Surgery and Surgical Oncology, National Cancer Centre Singapore, 169610 Singapore; 4 Division of Clinical Trails and Epidemiological Sciences, National Cancer Centre Singapore, 169610 Singapore

**Keywords:** caregivers, cancer, information sources, Internet, trust

## Abstract

Cancer impacts not only the patient but also the family members who share the distressing trajectory of the patient. The literature indicates that caregivers have many unmet information needs while providing care and support to the cancer patients, and caregivers have to resort to seeking information to supplement their information needs. This study aims to establish the prevalence of health-information-seeking behaviours among caregivers of cancer patients as a means of ascertaining if their information needs have been met and their information source and resource preference. Data were obtained via a self-reported questionnaire from caregivers of cancer patients at the National Cancer Centre Singapore between 10 September and 7 December 2018. A total of 986 caregivers responded of which 180 (18%) caregivers did not undertake information search and the common reasons were ‘trust healthcare professionals’ (HCPs) more than other sources (64%), and ‘HCPs provide enough information’ (59%). Among the 795 caregivers who have searched for cancer information, about half of these caregivers (54%) have searched information on the Internet and another 15% have obtained their information from HCPs in their most recent search. A total of 371 (47%) caregivers have used their preferred source of information to conduct their most recent information search. The top three most commonly sought information was treatment (35.6%), disease (35.6%) and side effects (26.5%). Almost half (46%) of these caregivers was concerned about the quality of information they have found on the Internet. Our study supports that information-seeking is prevalent amongst caregivers of cancer patients and reveals the prevalence of Internet use and the concerns associated with its use. Patterns of information-seeking revealed a discrepancy between preferred and actual source. The results also suggest that HCPs play a significant role in the information-seeking behaviours of caregivers of cancer patients.

## Background

Cancer impacts not only the patient but also the family members who share the distressing trajectory of the patient [1–3]. It disrupts family dynamics and changes roles and daily functioning [4]. Care responsibilities comprises both tangible assistance such as preparing meals for patients, providing transportation, helping with medication, communicating with doctors, and assessing the need for medication and treatment and intangible assistance, such as providing emotional, financial, social and spiritual support [5, 6]. Compounded to these is a shift responsibility for providing the tasks that often require a high degree of technical and observational skills that were previously provided by healthcare professionals (HCPs) to family members due to the shift in therapeutic management and treatment of cancer on an outpatient basis. Caring for patients with cancer is, therefore, a complex and demanding role and as such, caregivers of cancer patients experience a lot of distress [7–9] and burden [3, 5, 10, 11]. The distress and burden for caring for a person with cancer may be higher than Western society due to the Asian context where families are more involved in caring for one another. Asian society promotes social cohesion and interdependence where the family members are far more involved in caring for its members. Families are expected to care and support one another, especially the elderly, sick or disabled.

Caregiver needs have been well described in the literature and predominantly relate to information needs and psychological or emotional support [12–16]. Studies indicate that caregivers of cancer patients needed information on the disease, prognosis, treatment, and expected side effects and their management, hands-on care skills and accessing and navigating the healthcare system, including resources [5, 8, 12]. Information has been found to be helpful in assisting caregivers to cope by reducing the feeling of uncertainty [17]. However, these information needs are often unmet [7, 12–15, 18] and caregivers have to resort to seeking information to supplement their information needs [8, 16, 19]. The source of which family caregivers receive health information and the extent of how much they comprehend health information is crucial to achieving the best possible health outcomes as evidence revealed that they resorted to alternative mode of treatments to control symptoms and adverse effects of treatment [8] when their needs were unmet.

The research indicates that the Internet has been a common source for patients and caregivers to seek health information [20–25]. The common reasons cited for its use are convenience, amount of information available, immediacy of access, current and reliable information and privacy and anonymity [26, 27]. However, concerns have been raised about the quality of health information that is being posted online and whether information seekers possess the ability to effectively search, comprehend and discern the voluminous and highly variable quality of information [28, 29].

Since caregivers play a crucial role in supporting and caring for the cancer patient, and their ability to render care and support may be compromised by their lack of knowledge and skill, the role of health-information-seeking behaviour and resource preference have in caregivers’ needs can expand understanding of caregiver needs and is an essential step in achieving the goal of meeting their information needs. Such understanding can be used to inform the development of effective family caregiver education interventions to better deliver information in a manner that best meet their needs and preferences. Although studies have established the informational needs of caregivers of cancer patients; however, there was differing viewpoints on the type and amount of information they require [8, 12, 15, 18]. Besides, studies on information-seeking behaviours were mostly limited to the use of Internet [23–25], and there is a dearth of information on their information-seeking behaviours and their preferred source to receive information. As there is no reported study done on the information-seeking behaviours of caregivers of cancer patients in Singapore, this study seeks to establish the prevalence of health-information-seeking behaviours among caregivers of cancer patient and their resource preference in order to guide practice.

## Methods

### Study conduct and analysis

This study was a part of a larger survey on the unmet needs and quality of life of caregivers of cancer patients in Singapore. The study was conducted at the National Cancer Centre Singapore (NCCS) using the SPUNS-SF and the CQOLC-S25 between 10 September and 7 December 2018. The study population was caregivers of cancer patients who were defined as unpaid individuals who might be the parents, children, spouses, relatives or friends, providing one or more activities of daily living and healthcare needs for the cancer patients. Eligibility criteria for participation in the survey were: (1) able to read and/or write English or Mandarin and (2) the cancer patients they were providing care to have attended NCCS for at least 1 month. Exclusion criteria were: (1) domestic maids or helpers who were paid to take care of the patients and (2) caregivers of walk-in patients.

Data were collected by trained research assistants after confirmation of their eligibility criteria with the patients during their clinic visit. Consenting caregivers were invited to self-administer the questionnaire in a language of their preference (English or Chinese). The explanation was given about the purpose of the study, the voluntary nature and non-participation would not compromise the care and treatment of the patient, the anonymity of data collection and the procedures including how to fill up the questionnaire. For those patients whose caregivers met the eligibility criteria but did not accompany the patients during their clinic visit, the questionnaire with its explanatory note was given to the patients in a pre-paid envelope to bring home for the caregivers to complete. Ethical approval was obtained from the Centralized Institutional Review Board of the Singapore Health Services. Exemption from written consent was obtained as no identifiable data were collected.

Survey participants were those who had responded either yes or no to the question ‘Did you ever search for cancer information?’ in the questionnaire were included in this study. Participants who responded with missing response to this question were excluded from this study. Participants who responded that they have searched for information were further asked when was their most recent search, the actual source used during their search and the information sought, their most preferred source for cancer information and their experience with the information search.

Questions on information searching behaviours in the study were adapted based on a survey conducted by Hesse *et al* [30]. Modifications were made on the sources of the information that were commonly available in our local setting, and refinement was made based on our study aims including an open-ended question on the type of information sought. For caregivers who have not conducted any cancer information search, their reasons for not doing so were collected.

Demographics of respondents were also collected: age, sex, race, marital status, highest education level attained, economic status, monthly household income, housing type and relationship to cancer patient.

Descriptive statistics are used to summarise the characteristics of study participants and type of information searched for. Categorical characteristics were compared between the two groups of caregivers based on Fisher’s exact test. Continuous characteristics were compared using Mann-Whitney U test. All analyses were performed using SAS version 9.4 [31].

## Results

### Demographics of information seekers versus non-seekers

Compared with caregivers who have ever searched for cancer information, the non-searchers tended to be older (median: 40 versus 55 years; *p* < 0.001), had primary and below qualifications (2.9% versus 14%; *p* < 0.001) and residing in public Housing Development Board (HDB) 3-room or smaller flats (14% versus 23%; *p* < 0.001) (Table 1). A high percentage of caregivers who have ever searched for cancer information were children taking care of their parents with cancer (57% versus 25%; *p* < 0.001) (Table 1).

### Demographics of Internet information-seekers versus non-Internet seekers

Compared to caregivers who have ever searched for cancer information but have never used the Internet to conduct search, online seekers were younger (median: 47 versus 39 years; *p* < 0.001), had tertiary education (39% versus 58%; *p* < 0.001) and residing in public HDB 5-room flats or private housing (38% versus 50%; *p* = 0.05) (Table 2). A higher percentage of the non-online seekers had been taking care of their cancer patients for >5 years (42% versus 21%; *p* < 0.001) and not providing healthcare assistance to their patients (83% versus 69%; *p* = 0.025) (Table 2).

### Information-seeking patterns of caregivers

Of the 986 responded caregivers, 180 (18%) did not ever search for cancer information (Table 3). Common reasons why these caregivers did not undertake information search were ‘trust HCPs more than other sources’ (64%) and ‘HCPs provide enough information’ (59%) (Table 4).

### Source and preferred source of information

**Figure 1.**
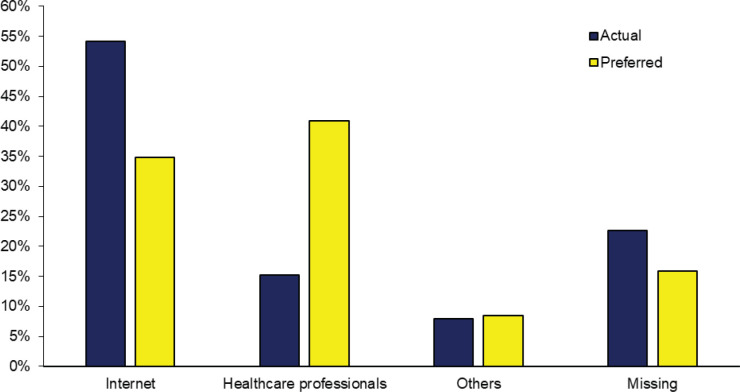
Source of information in most recent search.

Among the 795 caregivers who have ever searched for cancer information, about half of these caregivers (54%) have searched information on the Internet, and another 15% have obtained their information from HCPs (Figure 1). A total of 371 (47%) caregivers have used their preferred source of information to conduct their most recent information search. Among the 217 (27%) caregivers who did not use their preferred source, a large number (*n* = 164) of these caregivers’ preferred source was ‘HCPs’. While most caregivers generally had a good experience with their information search—42% disagreed that it took a lot of effort to get the information they needed, 47% disagreed that they were frustrated during the information search and 43% disagreed that the information found were too hard to understand, about 41% agreed that they were concerned about the quality of information they have found.

### Online health information-seeking

**Figure 2.**
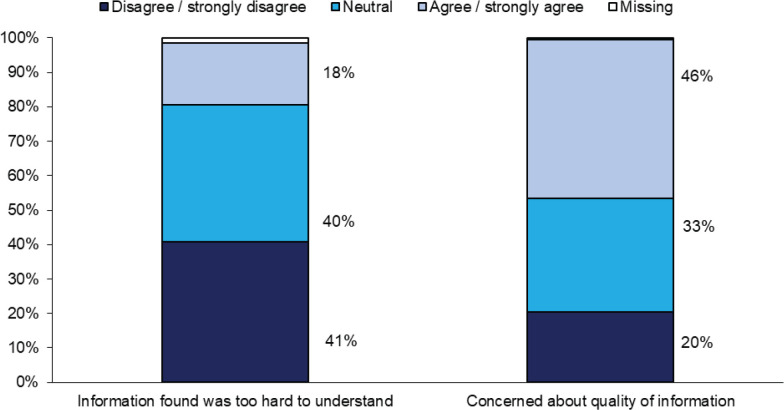
Internet search experience amongst searchers who have used Internet in last 1 year.

A high percentage of the 795 caregivers (87%) had used Internet to search for information about the disease of the patient they were taking care for in the last year prior to the survey. The main reason for the use of Internet by these caregivers was its convenience and accessibility (91%), and majority (71%) relied on search engines to search for the information they needed. A high percentage (46%) of these caregivers was concerned about the quality of information they have found on the Internet (Figure 2).

### Amongst caregivers with high or very high unmet needs by domains

Compared with overall cohort, there were a higher percentage of caregivers within each domain who agreed that they were concerned about the information found. The remaining information-seeking behaviours of caregivers with high or very high unmet needs in each domain of unmet needs were broadly similar as those of the overall cohort (Supplementary Tables A and B).

### Type of information searched

A free response question ‘What information did you look for?’ yielded 703 respondents. The top three topics being searched are treatment (35.6%), disease (35.6%) and side effects (26.5%) as shown in Table 5.

## Discussion

Information is essential for coping with cancer. The results of this survey support previous research that caregivers of cancer patients have information needs while providing care and support to the patients.

Health Information-seeking is prevalent amongst cancer caregivers. Only 18% did not search for cancer information and the main reasons were their trust in HCPs and they received adequate information from these professionals. Trust in HCPs was also cited by non-seekers in other studies [26, 32]. Hillen *et al* [33] reported that cancer patients trusted their physicians because the physicians were perceived to be technically competent, honest, displayed facilitative behaviours and had established a continuous relationship with them [33]. Trust is found to be associated with facilitation of the medical-decision making process, less worry about treatment, facilitate and improve treatment adherence and reduce the inclination to seek second opinion. Our study also revealed that non-searchers tended to be older, received lower education and residing in HDB 3-room or smaller flats. These flats are publicly developed by the government to provide affordable housing for the citizens. There are a variety of flat types which cater to different household sizes and budgets. This is in concordance with literature [19, 32, 34]. In Zilinski’s [34] review, cancer information non-seekers tended to be older, of lower income and received a lower level of education, and typically reported a high level of satisfaction and trust in doctor. Chen’s [19] study suggests that older caregivers tended to rely and trust HCPs due possibility to the authority of the information source.

Trust in HCPs may also be the reason why 40.9% of seekers preferred to receive information from them. The preference for HCPs is well reported in literature [16, 20, 30, 35, 36] as they are perceived to be the most trusted source to received health information [21, 37, 38]. However, only 15.2% of cancer caregivers were only able to obtain information from this preferred source. The accessibility of physicians may pose challenging for these caregivers due to the physicians’ busy schedule and heavy workload. Additionally, physicians are often unable to fully satisfy this desire for information because of the limited time available during clinical encounters especially so in an ambulatory care setting as evidenced by 25.5% of Internet information seekers gave that as one of the reasons. Our finding is supported by other studies [27, 35, 39]. Therefore, caregivers may have to resort to the Internet to obtain health information as evidenced that although 34.8% preferred to use the Internet, instead, 54.2% of caregivers actually used it. The finding that only 47% caregivers have used their preferred source to conduct their most recent information search indicates a mismatch and gap in service delivery.

Unlike Western culture which emphasises independence as a means to maintain self-esteem and to avoid becoming a burden to their children, in the Asian context, elderly parents look forward to having their children to provide and care for them. Children are brought up with the expectation to provide for and take care of their parents. As such it is not surprising that our study reveals more than half of the caregivers are children and they make up a high percentage of caregivers who have ever searched for cancer information. The majority of caregivers sought cancer specific information, namely, treatment, disease, and side effects and demonstrating the importance of such information as well as suggesting unmet information needs relating to the above topics. This finding is supported by literature [20, 35, 40–45]. Such information is needed to become more knowledgeable about the cancer, and, how best to help loved ones. Not having this information results in increased anxiety and stress for family caregivers [42]. As revealed, most of the health information sought for relates to the patients and only a small portion of caregivers sought health information for themselves. This may suggest that caregivers may have a tendency to neglect their own well-being while providing care and warrants further studies.

A high percentage of the caregivers (87%) had used Internet to search for information about the disease of the patient they were taking care for in the last year prior to the survey. Caregivers reported varying reasons and preferences for receiving information about cancer through the Internet. Besides being the gateway for an inexhaustible volume of information, convenience and accessibility is the top main reason for Internet use. Internet is readily accessible in Singapore as 91% of households have Internet access and 84% of individuals are Internet users [46] and can easily be found in homes, offices, schools, libraries and many other locations. Moreover, the widespread availability and usage of smartphones together with the proliferation of low-cost data plans have made the Internet more accessible. Our study also reveals that online seekers were younger, had tertiary education, and residing in HDB 5-room flats or private housing. This is supported by literature. Internet seekers are younger [30, 34, 38, 47], more educated [23, 30, 34, 38, 47], higher income/ higher economic status [30, 34, 38, 47] and Internet accessible at home [34]. Despite the availability of many health-related websites, the majority (71%) of caregivers relied on search engines to search for the information, with less than half using only established medical portal. Although increased access to health information can be helpful, the quality of information varies significantly between sources [49, 50]. The risk of using search engine is that a caregiver viewing that particular website may be influenced by its order of appearance on major search engines, with most web users only visited the top 10 websites listed in the search results [50]. Moreover, there is a lack of quality control of the medical and health information that is posted on the Internet, and anyone with access can establish a website and post medical information on the Internet [35]. There is also the variability of information with some being evidenced based while others can be unreliable and commercial in nature [28, 49]. This resulted in inconsistency in the quality of information being made available to the public. Even though 41.4% of caregivers claimed that the information found was not too hard to understand, they may not possess the scientific background needed to interpret the research/ information they retrieved. Besides, they may also lack the critical appraisal skills to distinguish the reliability of the webpage and if the information obtained is reliable [28].

This is a valid concern as reflected in our results that while most caregivers generally had a good experience with their information search, about 41% agreed that they were concerned about the quality of information they have found, with a high percentage (46%) of these caregivers was concerned about the quality of information they have found on the Internet. As caregivers are a primary source of support to cancer patients and are the first responders to changes in the patient’s status throughout each phase of the cancer care trajectory, they would require the knowledge and skills to care and support them. With limited access to HCPs to obtain information or opportunity to clarify information obtained from other sources, it is important to recognize this need and develop strategies to better support these caregivers. With the proliferation and readily access of information through the Internet and caregivers’ lack the needed skills to distinguish if the information obtained is reliable; besides making high-quality information available to both patients and their caregivers, strategies to mitigate the risks of unreliable information may include making available a list of high quality and accurate information web resources and establishing guidelines on how to evaluate health information from the Internet. In addition, in view of the diminished access to HCPs, strategies to address caregivers’ information needs also may include a more active role of the Cancer Helpline that is available within the institution.

## Limitations

Several limitations are inherent in this study. This is a cross-sectional study at a single point in time as we did not follow them over time as information needs may differ across cancer trajectory. The sample was also recruited from a single institution in Singapore which limits its ability to generalize to other settings and to all cancer caregivers. In addition, this report is a part of a larger study, and factors that may preclude a more comprehensive understanding may be excluded. Further studies should include the usefulness and trust of the information searched, reasons for the preference in the source, and the reasons why HCPs’ provision of information is inadequate. Notwithstanding these limitations, with the large sample size, it is, therefore, reasonable to assume that our results provide a reliable evidence of the information-seeking behaviours of caregivers of cancer patients and illuminates key needs and areas for improvement.

## Conclusion

The study concludes that caregivers of cancer patients are actively involved in information search indicating a need for information while providing care and support to the cancer patient. In addition, slightly more than a quarter of information searchers who used the Internet reason for doing so was the information provided by HCPs is insufficient. This implies that either additional or more detailed information to aid in learning and to assist in fulfilling their caregiving roles is needed. Less than half of caregivers have used their preferred source when conducting their most recent information search with the greatest mismatch being found in the HCPs. Caregivers indicated preferring to receive information from HCPs than informal resources such as the Internet. They resorted to the Internet to help address their information needs; however, they were concerned with the quality of information obtained. These further re-enforce the challenges confronting the caregivers and the support needed as they seek information to provide the needed care and support to the cancer patients. As caregivers play a crucial role in providing care and support to the cancer patient, and their ability to render care and support may be compromised by their lack of knowledge and skill, thus it is critical for HCPs to recognise, respect, assess, and address their information needs. It is also important to develop standardised information based on caregivers’ identified needs and deliver the information in various health formats so that information is readily available. Moreover, information delivery must also be in the manner and through the mode that is in accordance to their preference. In addition, given the high rates of Internet information-seeking, our finding suggests that this may be an ideal platform to deliver high-quality information, interventions, and reliable health related web-links for the younger, educated, and better off socioeconomically.

To the best of our knowledge, the present study is the first to examine the prevalence of health information-seeking of cancer caregivers and their preferred source for receiving information. The present study adds knowledge to the information-seeking behaviour of caregivers of cancer patients and the reasons for doing or not doing so.

## Conflicts of interest

The authors declare no conflict of interest.

## Funding declaration

This study was funded by the National Cancer Centre Research Fund (NCCRF-YR2018-JAN-PG6). The National Cancer Centre Research Fund has no role in the design, conduct and analysis of the study.

## Ethical consideration

Ethical consent was obtained from the SingHealth Centralised Institutional Review Board (CIRB) prior to the study. Waiver of written informed consent was obtained as no personal identifiers of respondents were obtained.

## Figures and Tables

**Table A.** Characteristics of searchers for cancer information.

Variable	Category	Total (*N* = 795)	Caregiver who reported high or very high unmet needs for ≥ 1 item in the domain of					
			‘I’ (*N* = 173)	‘P’ (*N* = 140)	‘E’ (*N* = 76)	‘W’ (*N* = 153)	‘C’ (*N* = 124)	‘F’ (*N* = 255)
		**No. (%)**	**No. (%)**	**No. (%)**	**No. (%)**	**No. (%)**	**No. (%)**	**No. (%)**
Most recent search	Past 1 month	333 (41.9)	100 (57.8)	85 (60.7)	51 (67.1)	68 (44.4)	67 (54.0)	138 (54.1)
	>1 to 6 months ago	192 (24.2)	41 (23.7)	38 (27.1)	19 (25.0)	45 (29.4)	35 (28.2)	67 (26.3)
	>6 to 12 months ago	87 (10.9)	14 (8.1)	6 (4.3)	4 (5.3)	14 (9.2)	11 (8.9)	16 (6.3)
	>12 months ago	170 (21.4)	15 (8.7)	10 (7.1)	2 (2.6)	23 (15.0)	9 (7.3)	31 (12.2)
	Missing	13 (1.6)	3 (1.7)	1 (0.7)	0 (-)	3 (2.0)	2 (1.6)	3 (1.2)
Actual source used in most recent search	Healthcare professionals	121 (15.2)	17 (9.8)	18 (12.9)	13 (17.1)	23 (15.0)	15 (12.1)	35 (13.7)
	Someone with cancer	16 (2.0)	6 (3.5)	3 (2.1)	3 (3.9)	4 (2.6)	3 (2.4)	10 (3.9)
	Books	4 (0.5)	2 (1.2)	0 (-)	1 (1.3)	1 (0.7)	0 (-)	1 (0.4)
	Cancer helplines	6 (0.8)	4 (2.3)	3 (2.1)	2 (2.6)	4 (2.6)	2 (1.6)	5 (2.0)
	Family	7 (0.9)	2 (1.2)	0 (-)	0 (-)	1 (0.7)	2 (1.6)	3 (1.2)
	Internet	431 (54.2)	76 (43.9)	66 (47.1)	28 (36.8)	68 (44.4)	55 (44.4)	121 (47.5)
	Magazines	2 (0.3)	0 (-)	1 (0.7)	1 (1.3)	0 (-)	0 (-)	1 (0.4)
	Library	5 (0.6)	1 (0.6)	0 (-)	0 (-)	0 (-)	0 (-)	0 (-)
	Friend/co-worker	15 (1.9)	7 (4.0)	4 (2.9)	3 (3.9)	4 (2.6)	4 (3.2)	7 (2.7)
	Brochures/ pamphlets	3 (0.4)	0 (-)	0 (-)	0 (-)	0 (-)	0 (-)	1 (0.4)
	Newspaper	2 (0.3)	0 (-)	1 (0.7)	1 (1.3)	1 (0.7)	0 (-)	1 (0.4)
	Others	3 (0.4)	0 (-)	0 (-)	0 (-)	0 (-)	0 (-)	1 (0.4)
	Missing	180 (22.6)	58 (33.5)	44 (31.4)	24 (31.6)	47 (30.7)	43 (34.7)	69 (27.1)
Preferred source	Healthcare professionals	325 (40.9)	59 (34.1)	54 (38.6)	29 (38.2)	57 (37.3)	48 (38.7)	100 (39.2)
	Someone with cancer	19 (2.4)	5 (2.9)	2 (1.4)	2 (2.6)	5 (3.3)	2 (1.6)	8 (3.1)
	Books	1 (0.1)	0 (-)	0 (-)	0 (-)	0 (-)	0 (-)	0 (-)
	Cancer helplines	14 (1.8)	7 (4.0)	4 (2.9)	1 (1.3)	6 (3.9)	5 (4.0)	10 (3.9)
	Family	6 (0.8)	2 (1.2)	0 (-)	0 (-)	1 (0.7)	2 (1.6)	1 (0.4)
	Internet	277 (34.8)	48 (27.7)	37 (26.4)	21 (27.6)	43 (28.1)	30 (24.2)	73 (28.6)
	Magazines	2 (0.3)	0 (-)	1 (0.7)	1 (1.3)	0 (-)	0 (-)	1 (0.4)
	Library	3 (0.4)	0 (-)	0 (-)	0 (-)	0 (-)	0 (-)	0 (-)
	Friend/co-worker	7 (0.9)	2 (1.2)	2 (1.4)	1 (1.3)	0 (-)	0 (-)	2 (0.8)
	Brochures/ pamphlets	8 (1.0)	3 (1.7)	2 (1.4)	0 (-)	3 (2.0)	1 (0.8)	3 (1.2)
	Newspaper	2 (0.3)	0 (-)	1 (0.7)	1 (1.3)	1 (0.7)	0 (-)	1 (0.4)
	Others	5 (0.6)	1 (0.6)	1 (0.7)	0 (-)	1 (0.7)	0 (-)	1 (0.4)
	Missing	126 (15.8)	46 (26.6)	36 (25.7)	20 (26.3)	36 (23.5)	36 (29.0)	55 (21.6)
Actual vs preferred source	Actual = preferred	**371 (46.7)**	61 (35.3)	54 (38.6)	30 (39.5)	61 (39.9)	41 (33.1)	106 (41.6)
	Actual ≠ preferred	**217 (27.3)**	46 (26.6)	36 (25.7)	19 (25.0)	39 (25.5)	34 (27.4)	69 (27.1)
	*Preferred = healthcare professionals*	**164 (20.6)**	34 (19.7)	29 (20.7)	15 (19.7)	30 (19.6)	27 (21.8)	53 (20.8)
	With ≥ 1 missing data for actual or preferred source	207 (26.0)	66 (38.2)	50 (35.7)	27 (35.5)	53 (34.6)	49 (39.5)	80 (31.4)
Information search experience:								
It took a lot of effort to get the info needed	Strongly disagreed	37 (4.7)	5 (2.9)	3 (2.1)	2 (2.6)	6 (3.9)	2 (1.6)	9 (3.5)
	Disagree	296 (37.2)	55 (31.8)	46 (32.9)	26 (34.2)	45 (29.4)	40 (32.3)	89 (34.9)
	Neutral	324 (40.8)	60 (34.7)	49 (35.0)	22 (28.9)	54 (35.3)	41 (33.1)	92 (36.1)
	Agree	104 (13.1)	40 (23.1)	33 (23.6)	25 (32.9)	37 (24.2)	32 (25.8)	51 (20.0)
	Strongly agree	16 (2.0)	9 (5.2)	6 (4.3)	0 (-)	8 (5.2)	7 (5.6)	11 (4.3)
	Missing	18 (2.3)	4 (2.3)	3 (2.1)	1 (1.3)	3 (2.0)	2 (1.6)	3 (1.2)
Felt frustrated during info search	Strongly disagreed	47 (5.9)	11 (6.4)	6 (4.3)	3 (3.9)	10 (6.5)	6 (4.8)	12 (4.7)
	Disagree	329 (41.4)	56 (32.4)	46 (32.9)	29 (38.2)	46 (30.1)	40 (32.3)	92 (36.1)
	Neutral	296 (37.2)	55 (31.8)	52 (37.1)	21 (27.6)	57 (37.3)	41 (33.1)	90 (35.3)
	Agree	91 (11.4)	40 (23.1)	28 (20.0)	19 (25.0)	33 (21.6)	28 (22.6)	49 (19.2)
	Strongly agree	14 (1.8)	7 (4.0)	5 (3.6)	3 (3.9)	4 (2.6)	7 (5.6)	9 (3.5)
	Missing	18 (2.3)	4 (2.3)	3 (2.1)	1 (1.3)	3 (2.0)	2 (1.6)	3 (1.2)
Info found was too hard to understand	Strongly disagreed	28 (3.5)	7 (4.0)	4 (2.9)	2 (2.6)	5 (3.3)	3 (2.4)	9 (3.5)
	Disagree	312 (39.2)	58 (33.5)	48 (34.3)	26 (34.2)	50 (32.7)	41 (33.1)	86 (33.7)
	Neutral	306 (38.5)	58 (33.5)	51 (36.4)	25 (32.9)	58 (37.9)	44 (35.5)	91 (35.7)
	Agree	113 (14.2)	38 (22.0)	27 (19.3)	19 (25.0)	31 (20.3)	28 (22.6)	56 (22.0)
	Strongly agree	18 (2.3)	8 (4.6)	7 (5.0)	3 (3.9)	6 (3.9)	6 (4.8)	10 (3.9)
	Missing	18 (2.3)	4 (2.3)	3 (2.1)	1 (1.3)	3 (2.0)	2 (1.6)	3 (1.2)
Concerned about quality of info	Strongly disagreed	21 (2.6)	6 (3.5)	2 (1.4)	1 (1.3)	3 (2.0)	3 (2.4)	5 (2.0)
	Disagree	168 (21.1)	22 (12.7)	17 (12.1)	7 (9.2)	19 (12.4)	20 (16.1)	33 (12.9)
	Neutral	265 (33.3)	35 (20.2)	29 (20.7)	17 (22.4)	45 (29.4)	23 (18.5)	59 (23.1)
	Agree	245 (30.8)	71 (41.0)	63 (45.0)	36 (47.4)	56 (36.6)	51 (41.1)	107 (42.0)
	Strongly agree	78 (9.8)	35 (20.2)	26 (18.6)	14 (18.4)	27 (17.6)	25 (20.2)	48 (18.8)
	Missing	18 (2.3)	4 (2.3)	3 (2.1)	1 (1.3)	3 (2.0)	2 (1.6)	3 (1.2)
Use internet to search info about patient’s disease in last 1 year	Never	82 (10.3)	12 (6.9)	7 (5.0)	6 (7.9)	8 (5.2)	7 (5.6)	20 (7.8)
	Rarely	86 (10.8)	14 (8.1)	7 (5.0)	6 (7.9)	14 (9.2)	12 (9.7)	19 (7.5)
	Sometimes	**339 (42.6)**	80 (46.2)	53 (37.9)	27 (35.5)	71 (46.4)	46 (37.1)	102 (40.0)
	Often	**269 (33.8)**	65 (37.6)	69 (49.3)	37 (48.7)	56 (36.6)	57 (46.0)	112 (43.9)
	Missing	19 (2.4)	2 (1.2)	4 (2.9)	0 (-)	4 (2.6)	2 (1.6)	2 (0.8)

I, information; P, personal; E, emotional; W, work & finance; C, access & continuity of healthcare; F, future.

**Table B.** Characteristics of searchers for cancer information who have used internet in last 1 year.

Variable	Category	Total (*N* = 694)	Caregiver who reported high or very high unmet needs for ≥ 1 item in the domain of					
			‘I’ (*N* = 159)	‘P’ (*N* = 129)	‘E’ (*N* = 70)	‘W’ (*N* = 141)	‘C’ (*N* = 115)	‘F’ (*N* = 233)
		No. (%)	No. (%)	No. (%)	No. (%)	No. (%)	No. (%)	No. (%)
Reason for using internet to search for info	Info provided by healthcare professionals is insufficient	**177 (25.5)**	67 (42.1)	54 (41.9)	32 (45.7)	53 (37.6)	51 (44.3)	91 (39.1)
	Convenience & accessible	**630 (90.8)**	138 (86.8)	114 (88.4)	62 (88.6)	125 (88.7)	98 (85.2)	198 (85.0)
	Protects privacy and keeps me anonymous	**107 (15.4)**	31 (19.5)	30 (23.3)	15 (21.4)	34 (24.1)	35 (30.4)	51 (21.9)
	Gateway to an inexhaustible volume of info	**272 (39.2)**	55 (34.6)	59 (45.7)	25 (35.7)	49 (34.8)	52 (45.2)	97 (41.6)
	Info provide is more current and reliable	**122 (17.6)**	32 (20.1)	26 (20.2)	16 (22.9)	31 (22.0)	22 (19.1)	51 (21.9)
	Others	16 (2.3)	7 (4.4)	6 (4.7)	4 (5.7)	6 (4.3)	6 (5.2)	8 (3.4)
Behaviour of internet search	Rely on search engines	**495 (71.3)**	112 (70.4)	87 (67.4)	47 (67.1)	97 (68.8)	80 (69.6)	156 (67.0)
	Browse only first few entries and modify search words till info needed is found	**225 (32.4)**	62 (39.0)	49 (38.0)	19 (27.1)	48 (34.0)	52 (45.2)	80 (34.3)
	Search through many sites	**270 (38.9)**	68 (42.8)	61 (47.3)	33 (47.1)	66 (46.8)	48 (41.7)	113 (48.5)
	Use only established medical portal	**218 (31.4)**	50 (31.4)	51 (39.5)	29 (41.4)	50 (35.5)	44 (38.3)	79 (33.9)
	Others	7 (1.0)	2 (1.3)	3 (2.3)	3 (4.3)	2 (1.4)	3 (2.6)	2 (0.9)
Information search experience:								
Info found was too hard to understand	Strongly disagreed	25 (3.6)	7 (4.4)	4 (3.1)	2 (2.9)	7 (5.0)	3 (2.6)	10 (4.3)
	Disagree	262 (37.8)	48 (30.2)	40 (31.0)	24 (34.3)	46 (32.6)	37 (32.2)	72 (30.9)
	Neutral	279 (40.2)	64 (40.3)	46 (35.7)	25 (35.7)	55 (39.0)	44 (38.3)	90 (38.6)
	Agree	**118 (17.0)**	37 (23.3)	35 (27.1)	18 (25.7)	28 (19.9)	26 (22.6)	55 (23.6)
	Strongly agree	**9 (1.3)**	2 (1.3)	3 (2.3)	0 (-)	4 (2.8)	4 (3.5)	5 (2.1)
	Missing	1 (0.1)	1 (0.6)	1 (0.8)	1 (1.4)	1 (0.7)	1 (0.9)	1 (0.4)
Concerned about quality of info	Strongly disagreed	14 (2.0)	2 (1.3)	1 (0.8)	0 (-)	2 (1.4)	1 (0.9)	3 (1.3)
	Disagree	127 (18.3)	17 (10.7)	16 (12.4)	10 (14.3)	20 (14.2)	17 (14.8)	27 (11.6)
	Neutral	230 (33.1)	44 (27.7)	25 (19.4)	17 (24.3)	41 (29.1)	24 (20.9)	58 (24.9)
	Agree	**246 (35.4)**	62 (39.0)	59 (45.7)	29 (41.4)	47 (33.3)	43 (37.4)	98 (42.1)
	Strongly agree	**74 (10.7)**	33 (20.8)	28 (21.7)	14 (20.0)	31 (22.0)	30 (26.1)	47 (20.2)
	Missing	3 (0.4)	1 (0.6)	1 (0.8)	0 (-)	0 (-)	0 (-)	0 (-)

I, information; P, personal; E, emotional; W, work & finance; C, access & continuity of healthcare; F, future.

**Table 1.** Characteristics of caregivers by whether caregiver has searched for cancer information.

Variable	Category	Searcher (*N* = 785)		Non-searcher (*N* = 180)		*p*-value
		No.	%	No.	%	
Age, years	≤30	179	22.5	34	18.9	<0.001
	>30–≤40	205	25.8	17	9.4	
	>40–≤50	202	25.4	21	11.7	
	>50–≤60	103	13.0	42	23.3	
	>60	48	6.0	49	27.2	
	Missing	58	7.3	17	9.4	
	Median (range)^1^	40 (14-76)		55 (18-84)		<0.001
Sex	Male	357	44.9	78	43.3	0.218
	Female	434	54.6	99	55.0	
	Missing	4	0.5	3	1.7	
Race	Chinese	581	73.1	130	72.2	0.930
	Malays	117	14.7	27	15.0	
	Indians	47	5.9	11	6.1	
	Others	35	4.4	7	3.9	
	Missing	15	1.9	5	2.8	
Marital status	Single	305	38.4	55	30.6	0.001
	Married	461	58.0	106	58.9	
	Widowed	4	0.5	5	2.8	
	Divorced/separated	16	2.0	6	3.3	
	Missing	9	1.1	8	4.4	
Highest education attained	No formal education	5	0.6	8	4.4	<0.001
	Primary	18	2.3	17	9.4	
	Secondary	79	9.9	50	27.8	
	Post-secondary	239	30.1	61	33.9	
	Tertiary	441	55.5	34	18.9	
	Missing	13	1.6	10	5.6	
Economic status	Full time	531	66.8	68	37.8	<0.001
	Part time	41	5.2	11	6.1	
	Schooling	46	5.8	17	9.4	
	Unemployed	36	4.5	11	6.1	
	Retired	46	5.8	32	17.8	
	Homemaker	51	6.4	23	12.8	
	Others	27	3.4	5	2.8	
	Missing	17	2.1	13	7.2	
Gross monthly household income, $	Not applicable	171	21.5	81	45.0	<0.001
	≤1,000	16	2.0	7	3.9	
	>1,000–≤2,000	34	4.3	3	1.7	
	>2,000–≤3,000	59	7.4	12	6.7	
	>3,000–≤4,000	83	10.4	18	10.0	
	>4,000–≤6,000	155	19.5	16	8.9	
	>6,000–≤8,000	93	11.7	8	4.4	
	>8,000–≤10,000	40	5.0	3	1.7	
	>10,000	65	8.2	7	3.9	
	Missing	79	9.9	25	13.9	
Housing type	HDB 1- & 2-room flats	11	1.4	7	3.9	<0.001
	HDB 3-room flats	100	12.6	34	18.9	
	HDB 4-room flats	273	34.3	51	28.3	
	HDB 5-room flats	214	26.9	43	23.9	
	Private housing	173	21.8	27	15.0	
	Missing	24	3.0	18	10.0	
Relationship to cancer patient	Parent	47	5.9	11	6.1	<0.001
	Child	451	56.7	45	25.0	
	Sibling	53	6.7	18	10.0	
	Spouse/partner	152	19.1	62	34.4	
	Friend	16	2.0	8	4.4	
	Others	63	7.9	30	16.7	
	Missing	13	1.6	6	3.3	
Living with cancer patient	Yes	496	62.4	107	59.4	0.011
	No	289	36.4	64	35.6	
	Missing	10	1.3	9	5.0	
No. of household members (among caregivers who lived with their patients)	Median (range)^1^	4 (1-10)		3 (1-8)		0.013
Type of caregiver	Primary	228	28.7	43	23.9	0.004
	Non-primary	544	68.4	122	67.8	
	Missing	23	2.9	15	8.3	
Duration of care, years	≤0.5	183	23.0	40	22.2	0.391
	>0.5–≤1	123	15.5	24	13.3	
	>1–≤3	176	22.1	33	18.3	
	>3–≤5	112	14.1	26	14.4	
	>5	183	23.0	49	27.2	
	Missing	18	2.3	8	4.4	
Type of care:						
Companionship	Yes	734	92.3	158	87.8	0.097
	No	46	5.8	15	8.3	
	Missing	15	1.9	7	3.9	
Transportation	Yes	632	79.5	122	67.8	0.003
	No	148	18.6	51	28.3	
	Missing	15	1.9	7	3.9	
Homemaking	Yes	353	44.4	74	41.1	0.214
	No	427	53.7	99	55.0	
	Missing	15	1.9	7	3.9	
Personal care assistance	Yes	104	13.1	19	10.6	0.186
	No	676	85.0	154	85.6	
	Missing	15	1.9	7	3.9	
Healthcare assistance	Yes	221	27.8	32	17.8	0.006
	No	559	70.3	141	78.3	
	Missing	15	1.9	7	3.9	
Financial assistance	Yes	414	52.1	51	28.3	<0.001
	No	366	46.0	122	67.8	
	Missing	15	1.9	7	3.9	
Others	Yes	5	0.6	2	1.1	0.163
	No	775	97.5	171	95.0	
	Missing	15	1.9	7	3.9	
Time spent on caregiving per week, hours	≤5	150	18.9	33	18.3	0.002
	>5–≤20	309	38.9	45	25.0	
	>20–≤40	122	15.3	37	20.6	
	>40	192	24.2	56	31.1	
	Missing	22	2.8	9	5.0	
Health status	Excellent	200	25.2	38	21.1	0.005
	Good	340	42.8	65	36.1	
	Satisfactory	223	28.1	58	32.2	
	Poor	13	1.6	9	5.0	
	Missing	19	2.4	10	5.6	
Impact of caregiving on health status	Made it better	45	5.7	4	2.2	0.050
	Did not affect it	661	83.1	148	82.2	
	Made it worse	67	8.4	18	10.0	
	Missing	22	2.8	10	5.6	

1 Among patients with non-missing data.

**Table 2.** Characteristics of caregivers who have searched for information by whether caregiver has used Internet for information search in the last 1 year.

Variable	Category	Yes (*N* = 694)		No (*N* = 82)		*p*-value
		No.	%	No.	%	
Age, years	≤30	169	24.4	8	9.8	<0.001
	>30–≤40	189	27.2	13	15.9	
	>40–≤50	178	25.6	21	25.6	
	>50–≤60	83	12.0	16	19.5	
	>60	31	4.5	14	17.1	
	Missing	44	6.3	10	12.2	
	Median (range)^1^	39 (14-73)		47 (18-76)		<0.001
Sex	Male	314	45.2	33	40.2	0.626
	Female	376	54.2	49	59.8	
	Missing	4	0.6	0	-	
Race	Chinese	510	73.5	60	73.2	0.025
	Malays	105	15.1	10	12.2	
	Indians	34	4.9	11	13.4	
	Others	32	4.6	1	1.2	
	Missing	13	1.9	0	-	
Marital status	Single	276	39.8	25	30.5	0.143
	Married	394	56.8	53	64.6	
	Widowed	3	0.4	1	1.2	
	Divorced/separated	15	2.2	1	1.2	
	Missing	6	0.9	2	2.4	
Highest education attained	No formal education	3	0.4	2	2.4	<0.001
	Primary	11	1.6	7	8.5	
	Secondary	58	8.4	16	19.5	
	Post-secondary	213	30.7	22	26.8	
	Tertiary	400	57.6	32	39.0	
	Missing	9	1.3	3	3.7	
Economic status	Full time	480	69.2	40	48.8	<0.001
	Part time	37	5.3	2	2.4	
	Schooling	44	6.3	2	2.4	
	Unemployed	30	4.3	6	7.3	
	Retired	35	5.0	10	12.2	
	Homemaker	31	4.5	18	22.0	
	Others	25	3.6	0	-	
	Missing	12	1.7	4	4.9	
Gross monthly household income, $	Not applicable	142	20.5	26	31.7	0.009
	≤1,000	14	2.0	1	1.2	
	>1,000–≤2,000	34	4.9	0	-	
	>2,000–≤3,000	52	7.5	6	7.3	
	>3,000–≤4,000	76	11.0	5	6.1	
	>4,000–≤6,000	136	19.6	13	15.9	
	>6,000–≤8,000	83	12.0	8	9.8	
	>8,000–≤10,000	38	5.5	1	1.2	
	>10,000	59	8.5	6	7.3	
	Missing	60	8.6	16	19.5	
Housing type	HDB 1- & 2-room flats	10	1.4	1	1.2	0.050
	HDB 3-room flats	92	13.3	8	9.8	
	HDB 4-room flats	227	32.7	36	43.9	
	HDB 5-room flats	192	27.7	18	22.0	
	Private housing	156	22.5	13	15.9	
	Missing	17	2.4	6	7.3	
Relationship to cancer patient	Parent	38	5.5	5	6.1	0.001
	Child	415	59.8	30	36.6	
	Sibling	43	6.2	7	8.5	
	Spouse/partner	115	16.6	31	37.8	
	Friend	15	2.2	1	1.2	
	Others	57	8.2	6	7.3	
	Missing	11	1.6	2	2.4	
Living with cancer patient	Yes	426	61.4	57	69.5	0.283
	No	259	37.3	24	29.3	
	Missing	9	1.3	1	1.2	
No. of household members (among caregivers who lived with their patients)	Median (range)^1^	4 (1–10)		4 (2–10)		0.586
Type of caregiver	Primary	199	28.7	23	28.0	1.000
	Non-primary	475	68.4	57	69.5	
	Missing	20	2.9	2	2.4	
Duration of care, years	≤0.5	174	25.1	7	8.5	<0.001
	>0.5–≤1	114	16.4	6	7.3	
	>1–≤3	157	22.6	14	17.1	
	>3–≤5	86	12.4	20	24.4	
	>5	147	21.2	34	41.5	
	Missing	16	2.3	1	1.2	
Type of care:						
Companionship	Yes	636	91.6	80	97.6	0.141
	No	44	6.3	1	1.2	
	Missing	14	2.0	1	1.2	
Transportation	Yes	548	79.0	68	82.9	0.815
	No	132	19.0	13	15.9	
	Missing	14	2.0	1	1.2	
Homemaking	Yes	306	44.1	38	46.3	0.933
	No	374	53.9	43	52.4	
	Missing	14	2.0	1	1.2	
Personal care assistance	Yes	89	12.8	11	13.4	0.955
	No	591	85.2	70	85.4	
	Missing	14	2.0	1	1.2	
Healthcare assistance	Yes	202	29.1	13	15.9	0.025
	No	478	68.9	68	82.9	
	Missing	14	2.0	1	1.2	
Financial assistance	Yes	363	52.3	40	48.8	0.770
	No	317	45.7	41	50.0	
	Missing	14	2.0	1	1.2	
Others	Yes	4	0.6	1	1.2	0.655
	No	676	97.4	80	97.6	
	Missing	14	2.0	1	1.2	
Time spent on caregiving per week, hours	≤5	132	19.0	13	15.9	0.025
	>5 - ≤20	278	40.1	22	26.8	
	>20 - ≤40	105	15.1	13	15.9	
	>40	159	22.9	32	39.0	
	Missing	20	2.9	2	2.4	
Health status	Excellent	178	25.6	21	25.6	0.335
	Good	304	43.8	29	35.4	
	Satisfactory	185	26.7	29	35.4	
	Poor	10	1.4	2	2.4	
	Missing	17	2.4	1	1.2	
Impact of caregiving on health status	Made it better	41	5.9	3	3.7	0.651
	Did not affect it	572	82.4	73	89.0	
	Made it worse	60	8.6	5	6.1	
	Missing	21	3.0	1	1.2	

1 Among patients with non-missing data.

**Table 3.** Ever search for cancer information.

	Total (*N* = 986)	Caregiver who reported high or very high unmet needs for ≥ 1 item in the domain of					
		‘I’(*N* = 199)	‘P’(*N* = 174)	‘E’(*N* = 92)	‘W’(*N* = 178)	‘C’(*N* = 146)	‘F’(*N* = 297)
	No. (%)	No. (%)	No. (%)	No. (%)	No. (%)	No. (%)	No. (%)
Yes	795 (80.6)	173 (86.9)	140 (80.5)	76 (82.6)	153 (86.0)	124 (84.9)	255 (85.9)
No	180 (18.3)	23 (11.6)	30 (17.2)	15 (16.3)	24 (13.5)	21 (14.4)	39 (13.1)
Missing	11 (1.1)	3 (1.5)	4 (2.3)	1 (1.1)	1 (0.6)	1 (0.7)	3 (1.0)

I, information; P, personal; E, emotional; W, work & finance; C, access & continuity of healthcare; F, future.

**Table 4.** Reason for not searching for cancer information among non-searchers.

	Total (*N* = 180)	Caregiver who reported high or very high unmet needs for ≥ 1 item in the domain of					
		‘I’(*N* = 23)	‘P’(*N* = 30)	‘E’(*N* = 15)	‘W’(*N* = 24)	‘C’(*N* = 21)	‘F’(*N* = 39)
	No. (%)	No. (%)	No. (%)	No. (%)	No. (%)	No. (%)	No. (%)
Healthcare professionals provide enough info	107 (59.4)	9 (39.1)	16 (53.3)	7 (46.7)	15 (62.5)	11 (52.4)	23 (59.0)
Trust healthcare professionals more than other sources	115 (63.9)	10 (43.5)	19 (63.3)	8 (53.3)	15 (62.5)	11 (52.4)	22 (56.4)
No computer	13 (7.2)	1 (4.3)	4 (13.3)	1 (6.7)	2 (8.3)	3 (14.3)	3 (7.7)
Have computer but no Internet access	2 (1.1)	0 (-)	1 (3.3)	0 (-)	0 (-)	0 (-)	0 (-)
Not acquainted with Internet	34 (18.9)	9 (39.1)	10 (33.3)	6 (40.0)	6 (25.0)	7 (33.3)	10 (25.6)
Others	32 (17.8)	6 (26.1)	6 (20.0)	1 (6.7)	3 (12.5)	4 (19.0)	9 (23.1)

I, information; P, personal; E, emotional; W, work & finance; C, access & continuity of healthcare; F, future.

**Table 5.** Type of information searched.

Category	Responses^1^
Treatment	250 (35.6%)
Disease	250 (35.6%)
Side effects	186 (26.5%)
Nutrition	177 (25.2%)
Cancer statistics	103 (14.7%)
Coping and support	97 (13.8%)
Complementary and alternative medicine	58 (8.3%)
Everything related to cancer	31 (4.4%)
Caregivers	29 (4.1%)
Others	54 (7.7%)

1 Total responses do not add to 703 as respondents may search for more than one type of information.
